# Effect of the Common Fat Mass and Obesity Associated Gene Variants on Obesity in Pakistani Population: A Case-Control Study

**DOI:** 10.1155/2015/852920

**Published:** 2015-08-18

**Authors:** Shabana, Shahida Hasnain

**Affiliations:** ^1^Department of Microbiology and Molecular Genetics, University of the Punjab, Lahore 54590, Pakistan; ^2^The Women University, Multan, Pakistan

## Abstract

*Background/Objective*. Obesity has become a global epidemic due to an increase in the number of obese individuals worldwide. There is little research in the field of obesity genetics in Pakistan. The aim of the current study was to analyze the association of common variants in Fat Mass and Obesity associated *(FTO)* gene with obesity in Pakistan, to find out the effect of the selected SNPs on anthropometric and biochemical traits, and to observe whether these variants act synergistically. *Methods*. Samples from 631 subjects were taken after informed consent and were used for serum parameters and genetic analysis. Lipid profile was determined, tetra-ARMS PCR was used for genotyping, and allele/genotype frequencies and genescore were calculated. *Results*. All *FTO* variants were associated with obesity, and some biochemical and anthropometric measures and had higher minor allele frequencies than those reported for Asian populations previously. The risk allele of each single nucleotide polymorphism resulted in an increase in BMI in a quantitative manner. *Conclusion*. Common forms of obesity are due to a combined net effect of many variants presented in same or different genes. The more the number of risk alleles present, the higher the risk and severity of obesity resulting from an increase in BMI.

## 1. Introduction

Obesity can be defined as abnormal or excessive fat accumulation that may impair health [1]. Obesity reports date as far back as Graeco-Roman times; however, major scientific progress in the field of obesity was not made until 20th century. It affects all age groups, adults as well as children [2]. World Health Organization (WHO) has designated obesity as global epidemic due to a dramatic increase in number of overweight and obese individuals worldwide [3]. It is now a well-established risk factor for mortality, cardiovascular diseases, diabetes, hypertension, dyslipidemia, gall bladder disease, and many others diseases [4]. It is strongly related to insulin resistance which is associated with blood lipid profile, blood pressure, and diabetes [5]. The children whose parents are obese tend to have high weight in the childhood and develop obesity as adults. Previously thought to be a problem of Western society mainly, obesity is now increasing in the developing world at an alarming rate as well [6]. A National Health Survey* (NHS 1990–1994)* reported that 5.2% of females and 1.6% of males in Pakistan over the age 15 are obese [7, 8].

Obesity is a heterogeneous disorder that involves a complex interaction of behavior, environment, and genetics [9]. Over the past 100 years, different biological and psychological explanations have been proposed to completely understand the pathophysiology and etiology of obesity [10]. Research to understand the genetic basis of obesity started with twin studies which indicated that 60–90% BMI variation in a population can be accounted for by genetic factors. It was observed that heritability estimates for twins reared apart are approximately the same as for twins reared together [11].

Body mass index (BMI, weight in kilograms divided by height in square meter) is conventionally used for classification of obesity and as a measure for tracking increase in the overall burden of obesity globally [12]. The use and value of BMI are, however, questioned in many studies because of ethnic differences in body weight distribution and due to the fact that it does not differentiate between fat mass and lean mass [12–14]. Relying solely on BMI for obesity classification may lead to under- or overestimating obesity [15]. Despite these limitations, its common use relies on the fact that it is a simple and easy measure that costs nothing and requires very little time, whereas other methods used to measure fat mass either are labor intensive, require expertise, or are expensive.

Identification of leptin gene mutations and treatment of obesity with recombinant leptin were milestone developments in the biomedical research of obesity and confirmed for the first time that single gene mutations can lead to obesity [16, 17]. A number of monogenic mutations in a variety of candidate genes were identified after this laying basis for monogenic obesity, but it soon became clear that these mutations are very rare. A key development in the field of obesity research was genome wide association studies (GWAS). After the first GWAS in 2007, a large number of variants in a variety of noncandidate genes have been identified that form the basis of common forms of polygenic obesity [18]. From such studies, it is now well established that genetic predisposition to common forms of obesity is largely due to combined net effect of polygenic variants [19].

Fat mass and Obesity associated (*FTO*) gene was the first gene discovered in 2007 in three independent genome wide association studies found to be associated with common forms of obesity [20–22].* FTO* gene spans more than 400 Kb on chromosome 16 and has nine exons. The first intron is most conserved and most of the variants identified to be associated with obesity are in the first intron [23]. The gene is well conserved across vertebrates and algae but not in invertebrates, plants, and fungi [24]. FTO protein is a 2-oxyglutarate dependent nonheme dioxygenase family member and localizes in nucleus [25, 26]. Recent rodent studies suggest that* FTO* is highly expressed not only in the hypothalamic nuclei involved in energy balance but also in the peripheral tissues [27].

As stated earlier, despite the large size of* FTO* gene, variants implicated in obesity and weight gain are largely located in the first intron. The association of common* FTO* variants, specially rs9939609, has been confirmed in Caucasians; however the results in Asian populations are somewhat conflicting [28–36]. Gender association of this SNP has also been reported previously, although not consistently [37]. A recent study in Pakistani population indicated the association of this SNP with female obesity and proposed that the SNP may play its role by affecting plasma glucose and leptin levels [38]. Due to this biological relevance and rationale, we aimed to replicate the findings of rs9939609 from previous studies and evaluate the effect of three other SNPs, rs8050136 [25, 35], rs1121980 [39], and rs9926289 [40], in the first intron of* FTO* reported to be related to obesity and correlate these variants with plasma lipid profile to elucidate the effect, if any, of these SNPs on lipid parameters.

## 2. Materials and Methods

### 2.1. Study Subjects

The study was carried out at the Department of Microbiology and Molecular Genetics, University of the Punjab, Lahore. A total of 631 subjects (obese = 346, controls = 285) were recruited by random sampling from hospitals and general population from Lahore, Sheikhupura, Bhakkar, and Burewala after obtaining written informed consent. The subjects were asked to fill a questionnaire regarding demographic information and family history of obesity.* The inclusion criteria for obese subjects were a body mass index (BMI) >30 Kg/m* ^*2*^ *and waist to hip ratio (WHR) ≥0.85 for women and ≥1 in men and for controls, the inclusion criteria were a BMI 18.5–24.99 while WHR <0.85 for women and <1 for men*. The BMI values to define and differentiate different categories of subjects were according to Asian cutoffs described by Mascie-Taylor and Goto, 2007 [41]. The exclusion criteria consisted of the presence of malignancies, pregnancy, and infections. All procedures were in compliance with Helsinki declaration and the study was approved by institutional ethics committee (Ethical Committee, School of Biological Sciences, University of the Punjab, SBS/273-17). Cases and controls were age and gender matched according to Cooper et al. [42].

### 2.2. Anthropometric Measurements

All anthropometric measurements were made according to standard procedures. Body weight, height, systolic and diastolic blood pressures, and waist and hip circumference were taken according to Shahid et al. BMI (body mass index) and WHR (waist to hip ratio) were calculated for each subject.

### 2.3. Blood Sampling

5 mL blood was drawn from the median cubital vein with tourniquet tied to the limb and fingers squeezed after 8–12 hr fasting. Blood from each individual was poured into two vacutainers 2.5 mL each, one containing EDTA to prevent blood clotting and the other containing a gel clot activator to accelerate blood clotting. Unclotted blood was used for DNA isolation while clotted blood was used to obtain plasma for determination of different biochemical parameters. For separating serum, gel vacutainers were centrifuged at 14,000 rpm for 10 min. Serum was collected in sterilized Eppendorf and used for biochemical measurements.

### 2.4. Biochemical Parameters

Glucose oxidase method was used to determine fasting blood glucose (FBG). Total cholesterol (TC), triglycerides (TG), high density lipoprotein cholesterol (HDL-c), and low density lipoprotein cholesterol (LDL-c) were measured using commercially available kits (Spectrum Diagnostics, Egypt). Epoch, Biotek microplate reader (Biotek instruments, Highland Park) was used for optical density measurements. VLDL-c was calculated from TG concentration (VLDL = TG/5). TC/HDL and LDL/HDL ratios were calculated.

### 2.5. Genotyping

Genomic DNA was isolated from whole blood manually using salting out method. Genotyping was done using tetra-ARMS (Allele Refractory Mutation detection System) PCR [43]. Primers were designed using primer designing program on http://primer1.soton.ac.uk/primer1.html. In order to validate the results of tetra-ARMS PCR, rs9939609 was also genotyped by PCR-RFLP according to Legry et al. [44]. Primers used for tetra-ARMS PCR and PCR-RFLP are shown in Supplementary Table  1 in Supplementary Material available online at http://dx.doi.org/10.1155/2015/852920. PCR reaction mixture (50 *µ*L) consisted of 200 ng DNA, 1X taq buffer, 200 *µ*M of each dNTP, 1 pmole of each outer primer, 10 pmole of each inner primer, 50 mM KCl, 20 mM tris HCl, 2.5 *µ*M MgCl_2_, and 0.5 U Taq polymerase. PCR program consisted of an initial denaturation at 95°C for 2 min, followed by 35 cycles of denaturation at 95°C for 1 min, annealing at 47.7°C for rs1121980, at 50.2°C for rs9939609, at 51.1°C for rs8050136, and at 52.8°C for rs9926289, extension at 72°C for 1 min, and a final extension at 72°C for 10 min. Advanced Primus 96 (PeqLab) thermal cycler was used for amplification. 105 bp amplified product for rs9939609 was digested with* Apo*I (Thermo Scientific, USA). PCR and restriction digestion products were run on 2% agarose gel to analyze the results.

### 2.6. Genetic Risk Score (GRS) Calculation

The genotypes were coded as 0, 1, and 2 for homozygous protective, heterozygous, and homozygous risk allele for each study subject and added up to get gene score. The effect size was calculated from the odds ratios and multiplied with total risk allele count, that is, gene score, to get a weighted risk score for each study subject. Average GRS for controls and cases was compared to the average population risk to determine the extent to which each group is having more risk of increased BMI and obesity related complications.

### 2.7. Statistical Analysis

For statistical analysis, statistical package for social sciences (IBM SPSS statistics, version 22) was used. Data was analyzed for mean, standard deviation, and normality for quantitative variables. Hardy-Weinberg equilibrium (HWE) test was applied to check whether both the cases and controls are in Hardy-Weinberg equilibrium. Allele/genotype frequencies were calculated from allele frequencies and chi-square test was applied to determine differences in allele and genotype frequencies between cases and controls. Odds ratio (OR) and 95% confidence intervals were calculated for each SNP. The association of risk alleles with obesity was determined using General Linear Model (GLM) assuming codominant, dominant, and recessive genetic models. Total number of risk alleles present in each individual in both cases and controls was determined and the mean risk of control and obese population as compared to the average population risk was calculated. To find out the effect of SNPs on anthropometric and biochemical traits, analysis of covariance was done after adjusting for covariates.

Logistic regression was applied to see the effect of the gene score on obesity and the association of SNPs with obesity.* As four SNPs were included in the study, present in the same gene, a corrected p value of 0.0125 was used as a significance cutoff*.

### 2.8. Linkage Disequilibrium Analysis of Variants

We analyzed the genotype data for all variants and tested them for linkage disequilibrium by SNP Annotation and Proxy Search (SNAP) program, Broad Institute. These SNPs have been reported to be in LD in previous studies but have not been confirmed in Pakistani obese subjects.

## 3. Results

General characteristics of the study population are given in Table 1. Cases consisted of 192 males and 154 females, whereas controls consisted of 139 males and 146 females. The case group had a higher percentage of comorbidities with 63 subjects suffering from hypertension, 80 from diabetes, and 30 from cardiovascular disorders, whereas in the control group there was 1 hypertensive, 3 diabetic, and no cardiac patient. Among the cases 159, while in controls, 4 subjects had a family history of obesity. The study population was in Hardy Weinberg equilibrium as tested by chi-square test. Chi-square test was also applied to test the significance of the distribution of risk alleles in cases and controls which came out to be significant for all SNPs (*p* < 0.05).* The genotyping success rates for rs9939609, rs9926289, rs1121980, and rs8050136 were 97.13%, 97.37%, 96.28%, and 98.11%, respectively, whereas the concordance rate for rs9939609 genotyped by two separate methods was 99.5%*.

### 3.1. Age and Gender Association

Study subjects were divided into quartiles (25th, IQR, and 75th percentile)* for age* and each was analyzed for association with each SNP one by one by *t*-test. We found a slightly different pattern of distribution of risk allele only for rs9939609 and not for other SNPs among adolescents and elderly subjects* (subjects between age 15 and 40 were considered adolescents while those >40 years of age were designated elderly)*. The frequency of A allele of rs9939609 was higher in subjects <40 years than those of age above this. Gender association was checked by independent sample *t*-test coding males as 1 and females as 2 but we found no significant association for gender in any of the SNPs.

### 3.2. Association of* FTO* SNPs with Obesity

The variants showed significant association with obesity as demonstrated by the differences in allele and genotype frequencies (Table 2). The odds ratio is greatest for rs9939609 followed by rs8050136, rs9926289, and rs1121980.* The minor allele frequencies (MAFs) observed in the current study are different compared to the previous reports whereby MAF observed was higher than previous studies for two SNPs, that is, 26.6% versus 12.1% for rs9939609 [45] and 43.5% versus 41.1 for rs8050136 [46], and lower for two SNPs, that is, 31.4% versus 43.1% for rs1121980 [46] and 30.5% versus 41.2% for rs9926289 [40]*.

### 3.3. Effect of* FTO* Variants on Lipid Profile

rs9939609 showed no association with any of the lipid profile parameters; however it was significantly associated with fasting blood glucose indicating its involvement in energy regulation pathway by exerting some effect on glucose metabolism. rs1121980 appeared to be associated with increased TC and TG levels (*p* < 0.001) and rs9926289 showed an increase in LDL (*p* = 0.014) and slight decrease in HDL (*p* < 0.05) whereas rs8050136 had no effect on lipid profile and FBG either (Table 3).

### 3.4. Association of* FTO* Variants with Anthropometric Parameters

Among the selected variants, rs9939609, rs9926289, and rs1121980 were significantly associated with BMI and waist circumference (*p* < 0.0125) but not with waist to hip ratio. rs8050136 was significantly associated with BMI and hip circumference only (*p* < 0.0125) with no effect on any other parameter (Table 3).

### 3.5. Combined Effect of Risk Alleles

**Figure 1:**
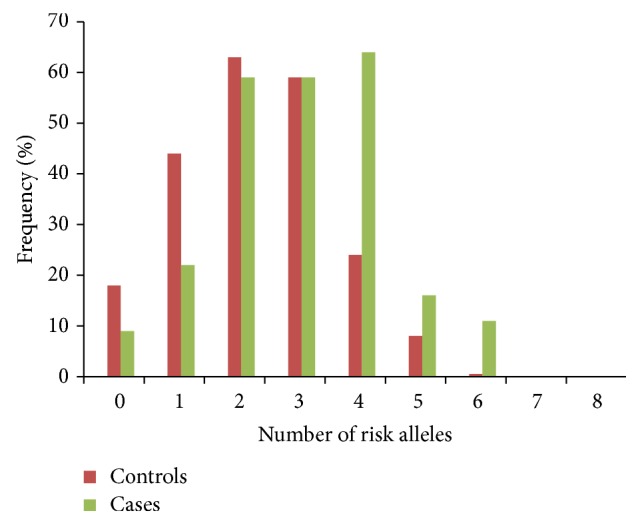
Frequency of subject carrying respective number of risk alleles in obese cases and nonobese control subjects. In cases, fewer individuals have <4 risk alleles, the majority have 4, and some have 6 risk alleles whereas fewer individuals have >3 risk alleles, the majority have <2, and very few have >4 risk alleles in controls.

**Figure 2:**
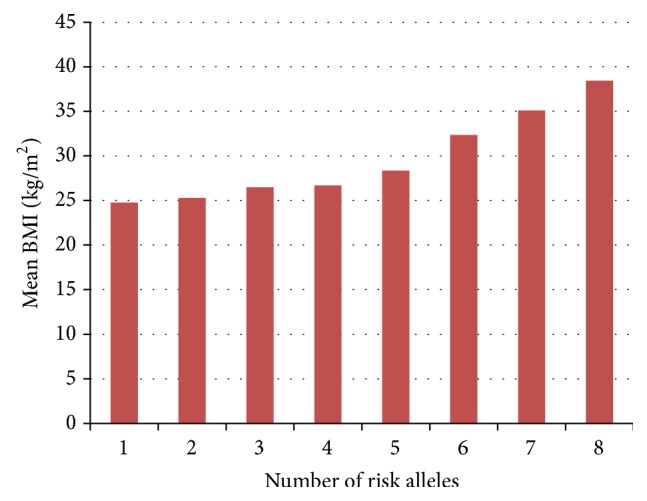
Effect of number of risk alleles on mean BMI. Bars indicate mean BMI values with respective number of risk alleles showing an additive effect of each risk allele to increase in mean BMI value.

Common forms of obesity involve a combined net effect of variants present in same or different genes [19]. To see whether this holds true for the* FTO* variants under study, we calculated the gene score for each individual in controls as well as cases (using 0, 1, and 2 coding for no, one, and two risk alleles) and plotted it for normal distribution in both groups* (Figure 1)*.* Independent sample t-test results showed a significance difference of mean gene score between cases and controls (2.99 ± 1.36 in cases versus 2.25 ± 1.25 in controls, p* = 3.16 × 10^−9^). In control group maximum individuals had a low number of risk alleles while in the obese group majority of subjects had a higher number of risk alleles. The normal distribution curve was shifted towards higher number of risk alleles in cases. The effect of the number of risk alleles on BMI was evaluated by plotting it against mean BMI. Mean BMI rather than exact values was used to get a more reliable plot as BMI varies with individuals, but mean value is a rather constant measure. Mean BMI appeared to increase as the number of risk alleles increases indicating the quantitative contribution of each allele to BMI increase* (Figure 2)*. The same effect was observed when relationship of risk score and BMI was analyzed. The control population had 17% higher GRS while cases had 47.89% higher GRS as compared to the average population risk.

### 3.6. Linkage Disequilibrium

All the variants were found to be in 100% LD with each other in Pakistani subjects for the first time indicating that these variants behave the same irrespective of the ethnicity. We manually cross-checked the genotype data and found consistent results, that is, same genotype combinations for all variants.

## 4. Discussion

The aim of the current study was to find the prevalence of common* FTO* variants in Pakistani population and hypothesize a role for each of them after observing the effect of these variants on serum lipid and glucose metabolism. rs9939609 is the most frequently studied intronic SNP of* FTO* but its frequency and association with obesity are controversial due to contradictory results among different population. All other SNPs are being studied in the Pakistani population for the first time. Our study showed the association of all these SNPs with obesity in Pakistani population and minor allele frequencies comparable to that in Caucasians. The fact that rs9939609 has no effect on lipid profile but has an effect on blood glucose suggests that it plays a role in energy regulation. The slight age association shows that its effects are more pronounced in adolescence as compared to childhood or old age. The pattern of association with anthropometric and biochemical parameter is in concordance with those previously reported [25, 39, 40]. A similar association for rs9939609 with obesity has been reported in East Asians and with type 2 diabetes in Indians [47–50]. Significant association of this variant with obesity in females <45 years and with type 2 diabetes in >40 year subjects has been reported in Pakistani population [38, 51]. We reported for the first time a complete linkage disequilibrium among these variants in Pakistani population.

In contrast to previous studies that reported low minor allele frequencies of rs9939609 in Asian population [52, 53] we found a higher MAF for rs9939609 and other variants comparable to those in European population [20]. The differences can be explained on the basis of BMI and WHR cutoffs used to categorize obesity, ethnicity, sample size, and behavioral and environmental factors. The high MAFs add power to the study and the associations observed.* The variant rs9939609 appeared to be slightly more prevalent in adolescents but the effect may be due to the fact that the subjects included in the study had a mean age of ~46 ± 15 years; therefore the presence of a large number of similar age individuals may have masked the actual association.*

The data were analyzed for correlation with cardiovascular disease indices included in the study (age, BMI, TC, and HDL-c). The presence of significant association for some variant with either of these indices provides a probable mechanism through which obesity may lead to cardiovascular disorders.

The study had several limitations. Firstly, the criteria used to categorize subjects were mainly BMI, which is questioned nowadays as a measure of obesity due to the fact that it does not distinguish between fat and lean mass. Other measures that are more accurate for estimating fat mass, for example, Dual-energy X-ray Absorptiometry (DEXA), could not be used due to high cost. Secondly, in order to draw reliable conclusions, the sample size should be quite large. Due to social and psychological stigma related to being overweight or obese, a large number of individuals refused to participate in the study. Thirdly, presence of comorbidities in the case group may have confounded certain associations. Although correlation analysis taking into account these comorbidities was performed, it may have affected results to some extent. Despite these limitations, the study did draw important conclusions about the behavior of the selected variants of a widely studied gene in the development of obesity. However, in future, larger studies should be performed in order to test for the reliability of the results of the current study.

## 5. Conclusion

The common* FTO* variants are associated with obesity in Pakistani population and exert their effect either by affecting energy regulation or altering lipid profile of the body. These variants also show an association with many anthropometric measures, indicating a role in fat deposition. Involvement in glucose metabolism and/or lipid profile thus predisposes to dyslipidemia, diabetes, cardiovascular diseases, and so forth. No gender and slight age association were observed. In future, more studies with larger sample size are needed to clarify the contradiction of age and gender association of* FTO* variants with obesity in Pakistani population.

## Supplementary Material

Supplementary Table 1: Primers and product sizes for tetra RMS and standard.

## Figures and Tables

**Table 1:** General characteristics of the study population.

Parameter	Obese (*n* = 346)	Controls (*n* = 285)	*p* value
Age (years)	40.63 (15.19)	39.78 (11.53)	0.436
Weight (Kg)	91.56 (16.05)	68.63 (10.23)	<0.0001
Height (ft)	5.36 (0.43)	5.4 (0.91)	0.468
BMI (Kg/m^2^)	34.37 (6.08)	22.67 (5.58)	<0.0001
WC (cm)	98.55 (12.11)	71.95 (8.65)	<0.0001
HC (cm)	105.51 (12.56)	78.23 (8.34)	<0.0001
WHR (WC/HC)	1.00 (0.07)	0.78 (0.01)	<0.0001
TC (mmol/L)	5.53 (0.90)	4.11 (0.79)	<0.0001
TG (mmol/L)	2.54 (0.83)	2.12 (0.05)	<0.0001
HDL-c (mmol/L)	1.11 (0.10)	2.09 (0.44)	<0.0001
LDL-c (mmol/L)	2.99 (0.56)	2.02 (0.34)	<0.0001
VLDL	0.51 (0.16)	0.42 (0.09)	<0.0001
TC/HDL	5.01 (0.97)	1.96 (0.15)	<0.0001
LDL/HDL	2.69 (0.36)	0.97 (0.06)	<0.0001
SBP (mmHg)	129.96 (5.9)	107.49 (1.19)	<0.0001
DBP (mmHg)	87.7 (0.92)	79.76 (0.98)	<0.0001
FBG (mg/dL)	102.95 (3.45)	87.56 (2.85)	<0.0001

Legend: Values for parameters are indicated as mean (SD) while *p* value indicates the significance of difference of the respective parameter between cases and controls.

**Table 2:** Allele/genotype frequencies in obese and control subjects.

SNP	Allele	Frequency (%)			Genotype	Frequency (%)		*p* value, OR (CI)
		Overall (*n* = 631)	Obese (*n* = 346)	Controls (*n* = 285)		Obese (*n* = 346)	Controls (*n* = 285)	
rs9939609	T **A**	73.4 26.6	70.7 29.3	76.5 23.5	TT TA AA	52.9 35.7 11.5	57.9 37.1 5.0	0.017 2.36 (1.14–4.86)

rs8050136	C **A**	56.5 43.5	52.9 47.1	63.6 36.4	CC CA AA	29.5 46.7 23.8	42.1 43.0 14.9	0.16 1.78 (1.11–2.85)

rs1121980	G **A**	68.6 31.4	62.5 37.5	75.3 24.7	GG GA AA	38.5 48.0 13.5	54.8 41.2 4.1	0.0003 3.68 (1.72–7.89)

rs9926289	G **A**	69.5 30.5	64.3 35.7	75.1 24.9	GG GA AA	41.8 45.1 13.1	56.6 37.1 6.3	0.014 2.23 (1.16–4.30)

Legend: OR: odds ratio, CI: confidence interval.

**Table 3:** Effect of selected SNPs on various anthropometric and biochemical parameters in the study subjects.

Parameter	rs9939609		rs8050136		rs1121980		rs9926289	
Weight	TT TA AA	73.91 ± 20.90 74.02 ± 20.79 81.74 ± 20.66	CC CA AA	71.39 ± 20.32 76.68 ± 21.25 75.55 ± 20.70	GG GA AA	71.95 ± 20.93 75.39 ± 20.41 84.31 ± 20.54	GG GA AA	71.85 ± 20.33 76.52 ± 21.84 80.20 ± 17.92

*β* per risk allele *p* value		0.079 0.084		0.081 0.075		0.041 0.102		0.133 0.004^*∗*^

BMI	TT TA AA	27.38 ± 6.36 29.01 ± 6.48 30.39 ± 5.77	CC CA AA	28.71 ± 6.31 29.76 ± 6.48 30.45 ± 6.15	GG GA AA	28.74 ± 6.27 29.58 ± 6.34 31.15 ± 6.58	GG GA AA	27.83 ± 5.56 30.10 ± 6.54 33.57 ± 6.90

*β* per risk allele *p* value		0.126 0.010^*∗*^		0.151 0.001^*∗*^		0.188 0.004^*∗*^		0.272 0.001^*∗*^

WC	TT TA AA	86.07 ± 12.42 87.09 ± 11.73 88.00 ± 12.79	CC CA AA	85.79 ± 11.20 87.22 ± 12.15 85.58 ± 13.50	GG GA AA	82.94 ± 11.85 84.70 ± 12.40 87.12 ± 12.68	GG GA AA	81.70 ± 12.03 86.45 ± 10.91 89.92 ± 16.22

*β* per risk allele *p* value		0.019 0.011^*∗*^		0.009 0.101		0.102 0.011^*∗*^		0.131 0.001^*∗*^

HC	TT TA AA	88.14 ± 13.07 87.27 ± 12.17 89.39 ± 12.45	CC CA AA	88.66 ± 12.07 87.37 ± 12.58 88.29 ± 13.62	GG GA AA	87.48 ± 12.36 88.62 ± 12.52 87.05 ± 14.14	GG GA AA	88.54 ± 12.49 87.32 ± 11.79 88.39 ± 15.95

*β* per risk allele *p* value		0.007 0.919		0.114 0.007^*∗*^		0.008 0.907		0.021 0.744

WHR	TT TA AA	1.00 ± 0.07 1.00 ± 0.08 0.98 ± 0.05	CC CA AA	0.99 ± 0.06 1.00 ± 0.07 1.01 ± 0.08	GG GA AA	1.01 ± 0.08 0.99 ± 0.06 1.01 ± 0.07	GG GA AA	1.01 ± 0.06 0.99 ± 0.07 1.01 ± 0.10

*β* per risk allele *p* value		0.059 0.362		0.063 0.33		0.058 0.37		0.02 0.761

Total Cholesterol (mmol/L)	TT TA AA	4.99 ± 1.08 5.09 ± 1.26 5.49 ± 1.09	CC CA AA	4.90 ± 1.05 5.15 ± 1.19 5.18 ± 1.22	GG GA AA	5.01 ± 1.16 5.10 ± 1.18 5.19 ± 1.01	GG GA AA	5.05 ± 1.26 5.04 ± 1.03 5.31 ± 1.09

*β* per risk allele *p* value		0.116 0.059		0.99 0.033		0.137 0.006^*∗*^		0.05 0.28

Triglycerides (mmol/L)	TT TA AA	2.30 ± 0.75 2.37 ± 0.94 2.63 ± 0.81	CC CA AA	2.36 ± 0.84 2.32 ± 0.76 2.39 ± 0.97	GG GA AA	2.31 ± 0.78 2.40 ± 0.90 2.32 ± 0.76	GG GA AA	2.30 ± 0.80 2.43 ± 0.91 2.28 ± 0.67

*β* per risk allele *p* value		0.099 0.031		0.011 0.809		0.125 0.009^*∗*^		0.033 0.472

HDL-c (mmol/L)	TT TA AA	1.44 ± 0.45 1.43 ± 0.50 1.29 ± 0.29	CC CA AA	1.50 ± 0.48 1.39 ± 0.45 1.36 ± 0.43	GG GA AA	1.49 ± 0.47 1.40 ± 0.46 1.22 ± 0.32	GG GA AA	1.47 ± 0.49 1.39 ± 0.43 1.30 ± 0.38

*β* per risk allele *p* value		−0.079 0.083		−0.013 0.111		−0.015 0.102		−0.116 0.011^*∗*^

LDL-c (mmol/L)	TT TA AA	2.56 ± 0.74 2.67 ± 0.74 2.76 ± 0.70	CC CA AA	2.48 ± 0.78 2.70 ± 0.71 2.67 ± 0.69	GG GA AA	2.61 ± 0.73 2.60 ± 0.77 2.75 ± 0.63	GG GA AA	2.54 ± 0.71 2.65 ± 0.77 2.84 ± 0.67

*β* per risk allele *p* value		0.098 0.034		0.009 0.112		0.019 0.677		0.122 0.008^*∗*^

FPG (mg/dL)		101.91 ± 13.52 103.21 ± 17.65 107.92 ± 16.54		98.45 ± 11.01 99.17 ± 14.75 99.07 ± 13.04		100.01 ± 10.52 99.24 ± 17.05 101.92 ± 13.14		102.41 ± 12.02 101.27 ± 16.25 102.12 ± 11.51

*β* per risk allele *p* value		0.198 0.006^*∗*^		0.019 0.112		0.019 0.277		0.002 0.208

Legend: Values are indicated as mean ± SD. BMI: body mass index, WC: waist circumference, HC: hip circumference, WHR: waist to hip ratio, FPG: fasting plasma glucose, HDL-c: high density lipoprotein cholesterol, LDL-c: low density lipoprotein cholesterol. *Analysis was done for cases and controls combined*.

^*∗*^indicates a significant association.

## References

[1] Hubbard V. S. (2000). Defining overweight and obesity: what are the issues?. *American Journal of Clinical Nutrition*.

[2] Eldin L. B., Hanan A., Soha E., Abou-Samra A. (2008). Relation between obesity, lipid profile, leptin and atopic disorders in children. *The Egyptian Journal of Pediatric Allergy and Immunology*.

[3] WHO Consultation (2000). *Obesity: Preventing and Managing the Global Epidemic*.

[4] Singh J., Thakur S. B. (2010). Blood lipid profile of obese and non obese sedentary college men. *VSRD Technical and Non Technical Journal*.

[5] Sinaiko A. R., Donahue R. P., Jacobs D. R., Prineas R. J. (1999). Relation of weight and rate of increase in weight during childhood and adolescence to body size, blood pressure, fasting insulin, and lipids in young adults: the Minneapolis children's blood pressure study. *Circulation*.

[6] Cummings D. E., Schwartz M. W. (2003). Genetics and pathophysiology of human obesity. *Annual Review of Medicine*.

[7] Chaudhry M. A., Ahmad F., Ashraf M. Z. (2012). Frequency of overweight and obesity in students of medical college of Lahore. *Annals of Pakistan Institute of Medical Sciences*.

[8] World Health Organization (2013). *WHO Global Infobase, Country Profiles*.

[9] Cheung W. W., Mao P. (2012). Recent advances in obesity: genetics and beyond. *ISRN Endocrinology*.

[10] Hebebrand J., Hinney A. (2009). Environmental and genetic risk factors in obesity. *Child and Adolescent Psychiatric Clinics of North America*.

[11] Hebebrand J., Wulftange H., Goerg T. (2000). Epidemic obesity: are genetic factors involved via increased rates of assortative mating?. *International Journal of Obesity*.

[12] Heber D. (2010). An integrative view of obesity. *American Journal of Clinical Nutrition*.

[13] Yusuf S., Hawken S., Ôunpuu S. (2005). Obesity and the risk of myocardial infarction in 27 000 participants from 52 countries: a case-control study. *The Lancet*.

[14] James W. P. T., Lobstein T. (2009). BMI screening and surveillance: an international perspective. *Pediatrics*.

[15] Heber D., Ingles S., Ashley J. M., Maxwell M. H., Lyons R. F., Elashoff R. M. (1996). Clinical detection of sarcopenic obesity by bioelectrical impedance analysis. *The American Journal of Clinical Nutrition*.

[16] Rankinen T., Zuberi A., Chagnon Y. C. (2006). The human obesity gene map: the 2005 update. *Obesity*.

[17] Gibson W. T., Farooqi I. S., Moreau M. (2004). Congenital leptin deficiency due to homozygosity for the Δ133G mutation: report of another case and evaluation of response to four years of leptin therapy. *Journal of Clinical Endocrinology and Metabolism*.

[18] Zhao J., Bradfield J. P., Zhang H. (2011). Role of BMI-associated loci identified in GWAS meta-analyses in the context of common childhood obesity in European Americans. *Obesity*.

[19] Hinney A., Vogel C. I. G., Hebebrand J. (2010). From monogenic to polygenic obesity: recent advances. *European Child & Adolescent Psychiatry*.

[20] Frayling T. M., Timpson N. J., Weedon M. N. (2007). A common variant in the FTO gene is associated with body mass index and predisposes to childhood and adult obesity. *Science*.

[21] Scuteri A., Sanna S., Chen W.-M. (2007). Genome-wide association scan shows genetic variants in the FTO gene are associated with obesity-related traits. *PLoS Genetics*.

[22] Dina C., Meyre D., Gallina S. (2007). Variation in FTO contributes to childhood obesity and severe adult obesity. *Nature Genetics*.

[23] Loos R. J. F., Bouchard C. (2008). FTO: the first gene contributing to common forms of human obesity. *Obesity Reviews*.

[24] Robbens S., Rouzé P., Cock J. M., Spring J., Worden A. Z., Van de Peer Y. (2008). The FTO gene, implicated in human obesity, is found only in vertebrates and marine algae. *Journal of Molecular Evolution*.

[25] Shing E. C., Tiwari A. K., Brandl E. J. (2014). Fat mass- and obesity-associated (FTO) gene and antipsychotic-induced weight gain: an association study. *Neuropsychobiology*.

[26] Gerken T., Girard C. A., Tung Y.-C. L. (2007). The obesity-associated FTO gene encodes a 2-oxoglutarate-dependent nucleic acid demethylase. *Science*.

[27] Fredriksson R., Hägglund M., Olszewski P. K. (2008). The obesity gene, FTO, is of ancient origin, up-regulated during food deprivation and expressed in neurons of feeding-related nuclei of the brain. *Endocrinology*.

[28] Price R. A., Li W.-D., Zhao H. (2008). FTO gene SNPs associated with extreme obesity in cases, controls and extremely discordant sister pairs. *BMC Medical Genetics*.

[29] Hinney A., Nguyen T. T., Scherag A. (2007). Genome Wide Association (GWA) study for early onset extreme obesity supports the role of fat mass and obesity associated gene (FTO) variants. *PLoS ONE*.

[30] Grant S. F. A., Li M., Bradfield J. P. (2008). Association analysis of the FTO gene with obesity in children of Caucasian and African ancestry reveals a common tagging SNP. *PLoS ONE*.

[31] Li H., Wu Y., Loos R. J. F. (2008). Variants in the fat mass- and obesity-associated (FTO) gene are not associated with obesity in a Chinese Han population. *Diabetes*.

[32] Ohashi J., Naka I., Kimura R. (2007). FTO polymorphisms in oceanic populations. *Journal of Human Genetics*.

[33] Hennig B. J., Fulford A. J., Sirugo G. (2009). FTO gene variation and measures of body mass in an African population. *BMC Medical Genetics*.

[34] Fang H., Li Y., Du S. (2010). Variant rs9939609 in the FTO gene is associated with body mass index among Chinese children. *BMC Medical Genetics*.

[35] Cha S. W., Choi S. M., Kim K. S. (2008). Replication of genetic effects of FTO polymorphisms on BMI in a Korean population. *Obesity*.

[36] Tan J. T., Dorajoo R., Seielstad M. (2008). FTO variants are associated with obesity in the chinese and malay populations in Singapore. *Diabetes*.

[37] Jacobsson J. A., Danielsson P., Svensson V. (2008). Major gender difference in association of FTO gene variant among severely obese children with obesity and obesity related phenotypes. *Biochemical and Biophysical Research Communications*.

[38] Shahid A., Rana S., Saeed S., Imran M., Afzal N., Mahmood S. (2013). Common variant of FTO Gene, rs9939609, and obesity in Pakistani females. *BioMed Research International*.

[39] Tran B., Nguyen N. D., Center J. R., Eisman J. A., Nguyen T. V. (2014). Association between fat-mass-and-obesity-associated (FTO) gene and hip fracture susceptibility. *Clinical Endocrinology*.

[40] Rosskopf D., Schwahn C., Neumann F. (2011). The growth hormone—IGF-I axis as a mediator for the association between FTO variants and body mass index: results of the Study of Health in Pomerania. *International Journal of Obesity*.

[41] Mascie-Taylor C. G. N., Goto R. (2007). Human variation and body mass index: a review of the universality of BMI cut-offs, gender and urban-rural differences, and secular changes. *Journal of Physiological Anthropology*.

[42] Cooper D. N., Nussbaum R. L., Krawczak M. (2002). Proposed guidelines for papers describing DNA polymorphism-disease associations. *Human Genetics*.

[43] Ye S., Dhillon S., Ke X., Collins A. R., Day I. N. (2001). An efficient procedure for genotyping single nucleotide polymorphisms. *Nucleic Acids Research*.

[44] Legry V., Cottel D., Ferrières J. (2009). Effect of an FTO polymorphism on fat mass, obesity, and type 2 diabetes mellitus in the French MONICA Study. *Metabolism*.

[45] Al-Attar S. A., Pollex R. L., Ban M. R. (2008). Association between the FTO rs9939609 polymorphism and the metabolic syndrome in a non-Caucasian multi-ethnic sample. *Cardiovascular Diabetology*.

[46] Shing E. C., Tiwari A. K., Brandl E. J. (2014). Fat mass-and obesity-associated (FTO) gene and antipsychotic-induced weight gain: an association study. *Neuropsychobiology*.

[47] Liu G., Zhu H., Lagou V. (2010). FTO variant rs9939609 is associated with body mass index and waist circumference, but not with energy intake or physical activity in European- and African-American youth. *BMC Medical Genetics*.

[48] Hotta K., Nakata Y., Matsuo T. (2008). Variations in the FTO gene are associated with severe obesity in the Japanese. *Journal of Human Genetics*.

[49] Yajnik C. S., Janipalli C. S., Bhaskar S. (2009). FTO gene variants are strongly associated with type 2 diabetes in South Asian Indians. *Diabetologia*.

[50] Sanghera D. K., Ortega L., Han S. (2008). Impact of nine common type 2 diabetes risk polymorphisms in Asian Indian Sikhs: PPARG2 (Pro12Ala), IGF2BP2, TCF7L2 and FTO variants confer a significant risk. *BMC Medical Genetics*.

[51] Rees S. D., Islam M., Hydrie M. Z. I. (2011). An FTO variant is associated with Type 2 diabetes in South Asian populations after accounting for body mass index and waist circumference. *Diabetic Medicine*.

[52] Xi B., Shen Y., Zhang M. (2010). The common rs9939609 variant of the fat mass and obesity-associated gene is associated with obesity risk in children and adolescents of Beijing, China. *BMC Medical Genetics*.

[53] Liu Y., Liu Z., Song Y. (2010). Meta-analysis added power to identify variants in FTO associated with type 2 diabetes and obesity in the Asian population. *Obesity*.

