# Supplementary material for: Effect of the Common Fat Mass and Obesity Associated Gene Variants on Obesity in Pakistani Population: A Case-Control Study
Source: Biomed Res Int. 2015 Aug 18;2015:852920. doi: 10.1155/2015/852920 (PMC4555445; doi:10.1155/2015/852920)
Supplement: Supplementary file 1 — Supplementary Table 1: Primers and product sizes for tetra RMS and standard. [file 852920.f1.pdf]

Sr no	SNP name	Primer	Sequence	Product
1	rs9939609	Forward Outer	CAGTTCCAGTCATTTTTGACAGC	Outer pair product: 446bp
		Reverse Outer	TGTTCAAGTCACACTCAGCCTCT	Product for A allele: 212bp
		Forward Inner	TCCTTGCGACTGCTGTGAATATA	Product for T allele: 148bp
		Reverse Inner	ACAGAGACTATCCAAGTGCATCTCA	
2	rs8050136	Forward Outer	GGGTTCATGAAGCCTCTGAAACT	Outer pair product: 428bp
		Reverse Outer	TTTCACACACCAAGATGGTCATG	Product for A allele: 277bp
		Forward Inner	AGTTGCCCACTGTGGCAGTC	Product for C allele: 196bp
		Reverse Inner	GCAAAAACCACAGGCTCAGATACTT	
3	rs1121980	Forward Outer	AAAAGCCAGATAAGGAGACTACTG	Outer pair product: 311bp
		Reverse Outer	GTGCCACCATATCTACCTCTTC	Product for T allele: 208bp
		Forward Inner	GCAGGTGGATCTGAAATCTAAT	Product for C allele: 148bp
		Reverse Inner	TAGTCACGTGTCTTGGTACTCTG	
4	rs9926289	Forward Outer	AAAAATTCAAAACTGGCTCTTGAATGAA	Outer pair product: 315bp
		Reverse Outer	GTCCAAACAGTAGGTCAGGAATAACCAG	Product for G allele: 208bp
		Forward Inner	CATTTAGAATGTCTGAATTATTATTCTCGG	Product for A allele: 163bp
		Reverse Inner	CAAAATTCACAGCAGTCGCAAGGCAT	
PCR-RFLP	rs9939609	Forward	GGTTCCTTGCGACTGCTGTGAAATT	105bp
		Reverse	GCTTTTATGCTCTCCCACTC	Restriction Enzyme: ApoI

Supplementary Table 1: Primers and product sizes for tetra RMS and standard
